# Comparison of Dorsal and Volar Percutaneous Approaches in Acute Scaphoid Fractures: A Meta-Analysis

**DOI:** 10.1371/journal.pone.0162779

**Published:** 2016-09-09

**Authors:** Kyu-Bok Kang, Hyun-Jung Kim, Jae-Hong Park, Young-Soo Shin

**Affiliations:** 1 Department of Orthopedic Surgery, Veterans Health Service Medical Center, Seoul, Korea; 2 Department of Preventive Medicine, Korea University College of Medicine, Seoul, Korea; BG Trauma Center Ludwigshafen, GERMANY

## Abstract

The dorsal approach allows better central screw placement along the long axis of the scaphoid compared with the volar approach in managing acute scaphoid fractures. However, it is unclear whether the dorsal approach leads to better clinical outcomes than the volar approach. This meta-analysis compared clinical outcomes, including the incidence of nonunion, postoperative complications, overall functional outcome, postoperative pain, grip strength, and range of wrist motion, between the dorsal and volar percutaneous approaches for the management of acute scaphoid fractures. Seven studies met the criteria for inclusion in the meta-analysis. The proportion of patients who developed nonunion (OR 0.74, 95% CI: 0.21 to 2.54; P = 0.63) and postoperative complications (OR 1.05, 95% CI: 0.45 to 2.44; P = 0.91) did not differ significantly between the dorsal and volar approaches. Both approaches also led to similar results in terms of overall functional outcome (95% CI: -0.39 to 0.22; P = 0.57), postoperative pain (95% CI: -0.52 to 0.46; P = 0.92), grip strength (95% CI: -4.56 to 1.02; P = 0.21), flexion (95% CI: -2.86 to 1.13; P = 0.40), extension (95% CI: -1.17 to 2.67; P = 0.44), and radial deviation (95% CI: -1.94 to 2.58; P = 0.78). However, ulnar deviation (95% CI: -7.48 to 0.05; P = 0.05) was significantly greater with the volar approach. Thus, orthopedic surgeons need to master both the dorsal and volar percutaneous approaches because not all acute scaphoid fractures can be dealt with completely with one approach.

## Introduction

The scaphoid is the most commonly fractured carpal bone, accounting for over 60% of carpal fractures and 11% of all hand fractures in young and active individuals.[1] Traditionally, nondisplaced or minimally displaced fractures involving the waist of the scaphoid have been treated by casting with thumb immobilization, but these methods require prolonged immobilization for at least 12 weeks, which may delay rehabilitation and lead to joint stiffness and poor clinical outcomes.[2,3] A trend in orthopedic practice toward open reduction and internal fixation for the treatment of fractures that have traditionally been treated conservatively can lead to early functional recovery by better fixation and more rapid bone union.[3–5] However, open reduction and internal fixation increase the risk of complications associated with damage to important structures, leading to carpal instability and tenuous vascular supply.[3,6] Thus, percutaneous screw fixation has increased in popularity with the use of new headless compression screws and better surgical techniques, for which the benefits offset the risks.[5] More recent studies comparing the dorsal and volar approaches have found better biomechanical and clinical outcomes.[7–10] Therefore, most orthopedic surgeons have shifted from open reduction and internal fixation to percutaneous screw fixation through the dorsal or volar approach. Several recent biomechanical studies have reported that central screw placement has been related to biomechanical superior outcome. Although central screw position can be more reliably achieved using a dorsal approach, it is unclear whether these biomechanical advantages are accompanied by clinical advantages.[9,11–13] In addition, no systematic reviews or meta-analyses have been published on this subject.

Therefore, this meta-analysis was designed to compare the clinical outcomes and complications of the dorsal and volar percutaneous approaches in patients undergoing surgical treatment of acute scaphoid fractures by evaluating the nonunion rate, postoperative complications, overall functional outcome, postoperative pain, grip strength, and range of wrist motion. It was hypothesized that the dorsal and volar approaches would show similar effectiveness and safety.

## Materials and Methods

This meta-analysis was conducted according to the guidelines of the preferred reporting items for systematic reviews and meta-analysis (PRISMA) statement (S1 Fig).

### Data and literature sources

This study followed the Cochrane Review Methods. Multiple comprehensive databases, including MEDLINE (January 1, 1976 to Dec 31, 2015), EMBASE (January 1, 1985 to Dec 31, 2015), WOS (January 1, 1980 to Dec 31, 2015), SCOPUS (January 1, 1980 to Dec 31, 2015), and the Cochrane Library (January 1, 1987 to Dec 31, 2015) were searched for studies that compared the nonunion rate, postoperative complications, overall functional outcome, postoperative pain, grip strength, and range of wrist motion after surgery in acute scaphoid fractures through the dorsal and volar percutaneous approaches. There were no restrictions on language or year of publication. Search terms used in the title, abstract, MeSH, and keywords fields included (‘scaphoid bone’ [Mesh] OR ‘fractures, bone’ [Mesh] OR ‘minimally invasive surgical procedures’ [Mesh] OR ‘dorsal approach’ [tiab] OR ‘volar approach’ [tiab]) AND ‘minimally invasive surgery’ [tiab] OR ‘scaphoid’ [tiab] OR ‘fractures’ [tiab] OR ‘percutaneous fixation’ [tiab]. After the initial electronic search, relevant articles and their bibliographies were searched manually.

### Study selection

From the title and abstract, two reviewers independently selected the relevant studies for full review. The full text copy of the article was reviewed if the abstract did not provide enough data to make a decision. Studies were included in the meta-analysis if they (1) assessed the nonunion rate, postoperative complications, overall functional outcome, postoperative pain, grip strength, and range of wrist motion after surgery in acute scaphoid fractures; (2) reported direct comparisons of surgical outcomes in acute scaphoid fractures through both the dorsal and volar percutaneous approaches; (3) included data on at least two of the following six parameters: nonunion rate, postoperative complications, overall functional outcome, postoperative pain, grip strength, and range of wrist motion, including flexion/extension, radial/ulnar deviation, or both. Overall functional outcome was based on validated hand and wrist function scores, including the Mayo wrist score system,[14] Disabilities of the Arm, Shoulder and Hand (DASH) score,[15] and Modified Mayo wrist score (MMWS).[16] Nonunion was defined as the absence of progressive fracture healing over 3 months at the fracture site and failure of the fracture to heal within 6 months of surgery, with radiographic evidence of a fracture line. A postoperative complication was defined as an adverse event of treatment recorded by the author of the study; (4) fully reported the number of patients in each group (dorsal and volar groups) and the means and standard deviations for the six parameters; and (5) used adequate statistical methods to compare these parameters between groups.

### Data extraction

Two reviewers independently recorded data from each study using a predefined data extraction form. Disagreement between the reviewers was resolved by consensus or by discussion with a third investigator when consensus could not be reached. Variables recorded included those associated with surgical outcomes, such as nonunion rate, postoperative complications, overall functional outcome, postoperative pain scale, grip strength, and range of wrist motion. Sample size and the means and standard deviations of surgical outcomes in each group were also recorded. If these variables were not included in the articles, the study authors were contacted by email to retrieve further information.

### Assessment of methodological quality

Two reviewers independently assessed the methodological quality of the studies. For prospective RCTs, methodological quality was assessed with the modified Jadad scale, which assesses randomization, blinding, withdrawals and dropouts, inclusion and exclusion criteria, adverse reactions, and statistical analysis. High quality studies have scores of 4–8, whereas low quality studies have scores of 0–3.[17] For the Newcastle-Ottawa Scale,[18] as recommended by the Cochrane Non-Randomized Studies Methods Working Group, we assessed the studies based on three criteria: selection of the study groups, comparability of the groups, and ascertainment of either the exposure or the outcome of interest for case-control and cohort studies. Any unresolved disagreements between reviewers were resolved by consensus or by consultation with a third investigator. Publication bias could not be assessed in these trials. Tests for funnel plot asymmetry are typically performed only when at least ten studies are included in the meta-analysis.[19] As our analysis included only seven studies, tests for asymmetry were not performed because these tests would not be able to differentiate asymmetry from chance.

### Data synthesis and analysis

The main outcomes of the meta-analysis were the proportion of cases that developed nonunion, postoperative complications, the weighted mean difference (WMD) in grip strength, and range of wrist motion; however, the standardized mean difference (SMD) was used for overall functional outcome and postoperative pain. For all comparisons, odds ratios (ORs) and 95% confidence intervals (CI) were calculated for binary outcomes, while WMD or SMD and 95% CI were calculated for continuous outcomes. Grip strength measurements were determined as a percentage of the value for the unaffected side in three studies and as kilograms in one study. When the percentage compared with the unaffected side was provided, data were standardized with equal weighting of the kilograms according to the outcome measures from a previous study.[8,10] Range of wrist motion measurements were determined as a percentage of the value for the unaffected side in two studies and as degrees in four studies. By using the same method described above, data were standardized by equal weighting of the degrees. For the overall functional outcome measure, we combined comparable scores from different functional outcome tools when these tools scored disability on a 100-point scale; the lower the score, the greater the disability. Using the same method, we combined comparable scores of postoperative pain as presented on a 100-point scale, where 0 indicates absence of pain and 100 indicates the worst pain imaginable. When standard deviations (SDs) were not included, we calculated the SDs from the confidence interval (CI) or *P* value.[19] Heterogeneity was determined by estimating the proportion of between-study inconsistencies due to actual differences between studies, rather than differences due to random error or chance using the I^2^ statistic, with values of 25%, 50%, and 75% considered low, moderate, and high heterogeneity, respectively. All statistical analyses were performed with RevMan version 5.2 static software. Subgroup analysis was only performed for range of wrist motion in an attempt to explore a potential source of heterogeneity. As a result, four subgroups were created (flexion, extension, radial deviation, and ulnar deviation). In addition, sensitivity analysis was performed by excluding one of the eligible studies at a time; 3 studies with Herbert type B3 were included.[8,16,20] Pooling of data was feasible for only two outcomes of interest, i.e. nonunion and complication rates.

## Results

### Identification of studies

**Fig 1:**
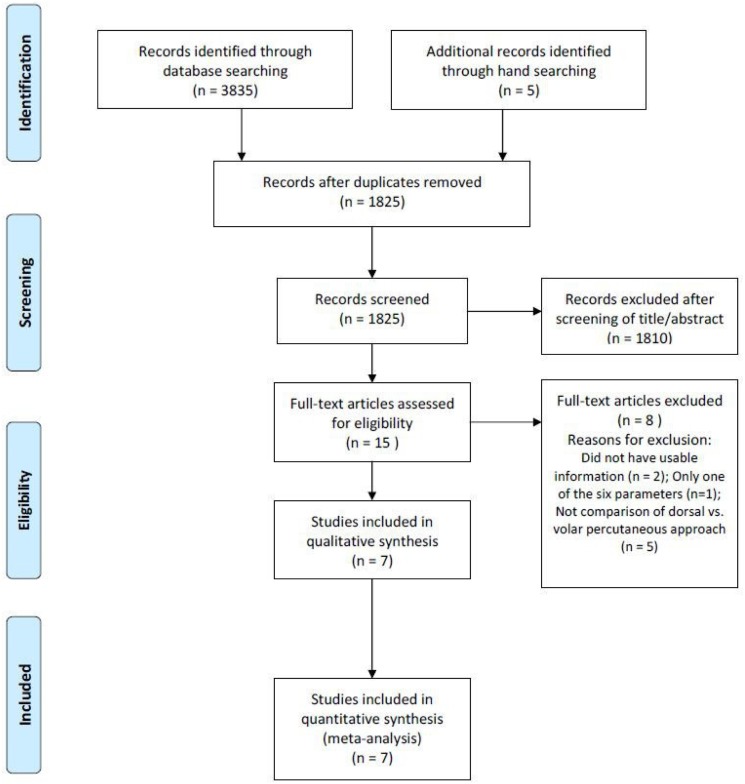
Preferred reporting items for systematic reviews and meta-analyses (PRISMA) flow diagram of literature selection.

The details on study identification, inclusion, and exclusion are summarized in Fig 1. An electronic search yielded 924 studies in PubMed (MEDLINE), 1119 in EMBASE, 640 in WOS, 1120 in SCOPUS, and 32 in the Cochrane Library. Five additional publications were identified through manual searching. After removing 2015 duplicates, 1825 studies remained; of these, 1810 were excluded based on reading the abstracts and full-text articles, and an additional 8 studies were excluded because they had unusable information, measured only one of the six parameters (i.e., nonunion rate, postoperative complications, overall functional outcome, postoperative pain, grip strength, or range of wrist motion), or made inappropriate group comparisons. This eventually resulted in 7 studies that were included in the meta-analysis.[7,8,16,20–23].

### Study characteristics and patient populations

The 7 studies we examined included 141 patients who underwent surgical treatment of acute scaphoid fractures through the dorsal percutaneous approach and 142 patients who underwent surgical treatment of acute scaphoid fractures through the volar percutaneous approach. Two studies (1 RCT and 1 PCS) compared prospectively measured parameters, whereas the other five studies compared parameters measured by retrospective chart review. Six studies compared the nonunion rate and range of wrist motion, seven compared postoperative complications, two compared the postoperative pain scale, five compared the overall functional outcome, and four compared grip strength (Table 1).

**Table 1:** Characteristics of the studies included in the meta-analysis.

Study	Year	Study type	Sample size		Herbert type	Method of treatment	Follow-up (months)	Quality score	Measured parameters
			Dorsal	Volar					
Drac et al.[23]	2010	RCS	38	42	A2, B2	HC	At least 12	7	NUR, ROWM, GS, POC
Drac et al.[21]	2012	RCT	37	37	B2	HC	At least 12	5	NUR, OFO, ROWM, GS, POC
Gürbüz et al.[8]	2012	RCS	13	14	B1, B2, B3	HCVPCS	At least 37	7	NUR, OFO, ROWM, GS, POC
Jeon et al.[7]	2009	PCS	22	19	B2	HC	Mean 30	8	NUR, OFO, POP, ROWM, POC
Parajuli et al.[16]	2012	RCS	2	13	A2, B2, B3, C	HC	Mean 24	8	NUR, OFO, POC
Polsky et al.[22]	2002	RCS	16	10	B2	CDPCS	At least 14	7	NUR, POP, ROWM, GS, POC
Slade et al.[20]	2008	RCS	13	7	B2, B3	HVPS	Mean 18	7	OFO, ROWM, POC

Abbreviations: RCS, retrospective comparative study; RCT, randomized controlled trial; PCS, prospective comparative study; HC, Herbert screw; HCVPCS, headless cannulated variable pitch compression screw; CDPCS, cannulated differential pitch compression screw; HVPS, headless variable pitch screw; NUR, nonunion rate; OFO, overall functional outcome; POP, postoperative pain; ROWM, range of wrist motion; GS, grip strength; POC, postoperative complications

### Methodological quality assessment of the included studies

The quality of the seven studies included in the meta-analysis is summarized in Table 1. There was one RCT of high quality (modified Jadad scale > 4). The non-RCTs (one PCS and five RCS) were of high quality (Newcastle-Ottawa Scale > 7).

### Nonunion and postoperative complication rates

**Fig 2:**
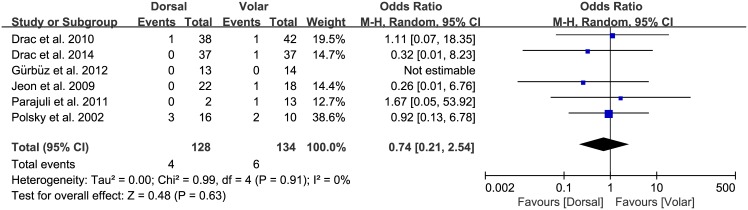
Results of aggregate analysis for comparison of nonunion rate (NUR) according to different approaches of acute scaphoid fracture.

**Fig 3:**
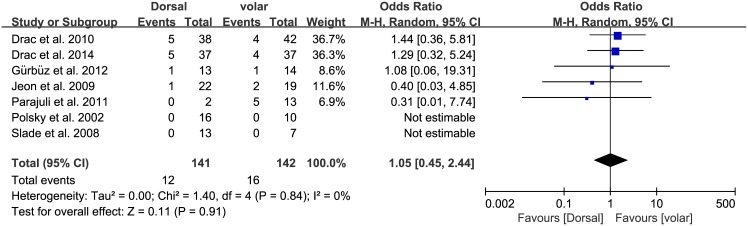
Results of aggregate analysis for comparison of postoperative complications (POC) according to different approaches of acute scaphoid fracture.

**Fig 4:**
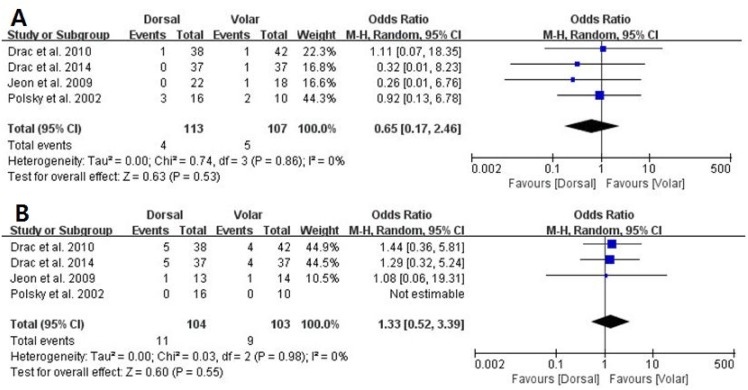
Sensitivity analysis. Forest plots of: (A) nonunion rate (NUR) and (B) postoperative complications (POC) for studies with Hertbert type B3.

Of the seven studies, six compared the nonunion rate in the dorsal and volar groups, which consisted of 128 and 134 patients, respectively. The proportion of patients who developed nonunion was similar between groups (dorsal, 4/128; volar, 6/134; OR 0.74, 95% CI: 0.21 to 2.54; P = 0.63; I^2^ = 0%, Fig 2). All seven studies presented data on the proportion of patients who developed postoperative complications, with no significant difference between groups (dorsal, 12/141; volar, 16/142; OR 1.05, 95% CI: 0.45 to 2.44; P = 0.91; I^2^ = 0%, Fig 3). The results of sensitivity analysis were not materially differentiated compared with those of the original analysis, including that the findings are robust to the decisions made in the process of obtaining them (Fig 4A and 4B).

### Overall functional outcome, postoperative pain, and grip strength

**Fig 5:**
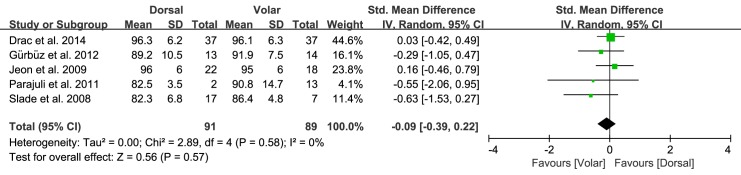
Results of aggregate analysis for comparison of overall functional outcome (OFO) according to different approaches of acute scaphoid fracture.

**Fig 6:**
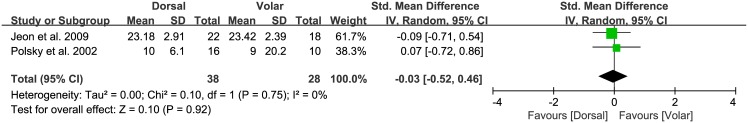
Results of aggregate analysis for comparison of postoperative pain (POP) according to different approaches of acute scaphoid fracture.

**Fig 7:**
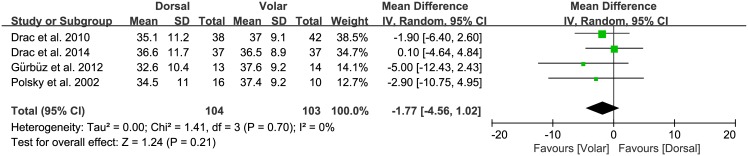
Results of aggregate analysis for comparison of grip strength (GS) according to different approaches of acute scaphoid fracture.

Of the seven studies, five compared the overall functional outcome for the two approaches, involving 91 patients treated with the dorsal approach and 89 treated with the volar approach. The standardized mean was 0.09 points lower in the dorsal group than the volar group, but this difference was not significant (95% CI: -0.39 to 0.22 points; P = 0.57; I^2^ = 0%, Fig 5). Two studies, including 38 patients treated with the dorsal approach and 28 treated with the volar approach, reported the postoperative pain. The standardized mean was 0.03 points lower with the dorsal group than the volar group, but this difference was not significant (95% CI: -0.52 to 0.46 points; P = 0.92; I^2^ = 0%, Fig 6). Four studies compared grip strength between the two approaches, involving 104 patients treated with the dorsal approach and 103 treated with the volar approach. The pooled data showed that mean grip strength was 1.77 kg greater with the volar approach than the dorsal approach, but this difference was not significant (95% CI: -4.56 to 1.02 kg; P = 0.21; I^2^ = 0%, Fig 7).

### Range of wrist motion (flexion, extension, radial deviation, and ulnar deviation)

**Fig 8:**
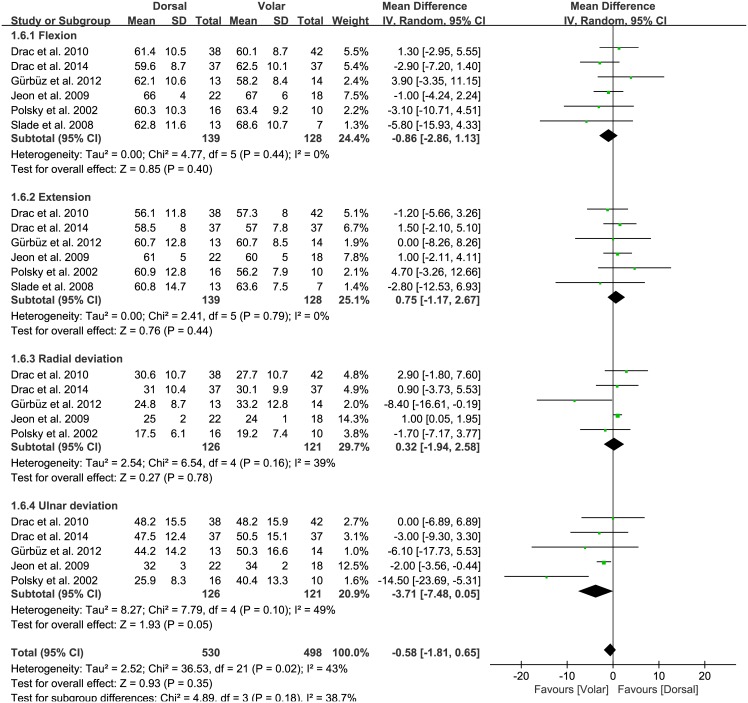
Results of aggregate analysis for range of wrist motion (ROWM) according to different approaches of acute scaphoid fracture, including subgroup analysis by flexion, extension, radial deviation, and ulnar deviation.

Of the seven studies, six compared the range of wrist motion between the two groups. The pooled mean difference in the range of wrist motion was -0.58 degrees (95% CI: -1.81 to 0.65 degrees; P = 0.18; I^2^ = 43%, Fig 8), with no significant difference between the dorsal and volar groups. Six studies were included in the flexion/extension subgroups, and five were included in the radial/ulnar deviation subgroups. For the flexion subgroup, the volar approach led to 0.86 degrees greater flexion than the dorsal approach, but this difference was not significant (95% CI: -2.86 to 1.13 degrees; P = 0.40; I^2^ = 0%, Fig 8). For the extension subgroup, the dorsal approach led to 0.75 degrees greater extension than the volar approach, although this difference was not significant (95% CI: -1.17 to 2.67 degrees; P = 0.44; I^2^ = 0%, Fig 8). For radial deviation, the dorsal approach led to 0.32 degrees greater radial deviation than the volar approach, another difference that was not significant (95% CI: -1.94 to 2.58 degrees; P = 0.78; I^2^ = 39%, Fig 8). In contrast, the pooled mean difference in the ulnar deviation subgroup was -3.71 degrees (95% CI: -7.48 to 0.05 degrees; P = 0.05; I^2^ = 49%, Fig 8), indicating that ulnar deviation was significantly greater for the volar than the dorsal approach.

## Discussion

The most important finding of this meta-analysis was that the dorsal and volar approaches for the surgical treatment of acute scaphoid fracture did not lead to significant differences in the nonunion rate, postoperative complications, overall functional outcome, postoperative pain, grip strength, or range of wrist motion, including flexion, extension, or radial deviation. However, the volar approach led to significantly greater ulnar deviation than the dorsal approach.

Percutaneous treatment of scaphoid fractures through the dorsal and volar approaches has been shown to yield better results than open techniques.[7,16,24] For example, one study reported that the required division of the volar radiocarpal ligaments or dorsal capsular structures was increased by open exposure of the scaphoid compared with percutaneous screw fixation.[6] Theoretically, the dorsal approach could lead to better clinical outcomes than the volar approach in acute scaphoid fractures because the dorsal approach allows central screw placement through the scaphoid, yielding greater stiffness with secure fixation and a lower risk of screw failure than eccentrically placed screws.[9] A cadaveric study that compared screw placement between the two approaches found that the dorsal approach achieved better central screw placement along the long axis of the scaphoid compared with the volar approach, suggesting that the dorsal approach may provide greater stability than the volar approach in managing acute scaphoid fractures.[11] Furthermore, fixation of proximal pole fractures is easier using the dorsal approach due to better operative exposure, and the dorsal approach avoids damage to the volar radiocarpal ligament, thus providing more favorable conditions for fracture healing by maintaining carpal kinematics.[25] In contrast, the volar approach requires the guide wire to be placed at the level of the scaphotrapezial joint, which can lead to volar placement of the screw in the distal pole of the scaphoid due to the volar surface of the trapezium.[26–28] It can also disrupt the superficial palmar arch and the recurrent branch of the median nerve, even when using a safe zone for volar guide wire insertion for the scaphoid screw based on radiographs, computed tomography (CT), and anatomical dissections.[11,29,30] These situations may interrupt bone healing by violating the volar blood supply to the distal pole, increasing the risk of nonunion with the volar approach, even though main blood supply around the scaphoid is dorsal carpal branch of the radial artery.[29,31]

Although the dorsal approach allows a surgeon to put the screw along the long axis of the scaphoid more reliable, the volar approach may be preferable to the dorsal approach because of the clinical limitations of dorsal approach. Many studies have reported complications associated with the dorsal approach, with incidences ranging from 0% to 29%.[32,33] With the dorsal approach, it is necessary to avoid placing any screw in a prominent position proximally that could lead to injury to the joint surface of the scaphoid fossa of the radius because of the shape of the proximal pole of the scaphoid and the design of current screws with a broader trailing head. This could result in shorter screws which could affect the fixation strength.[34,35] In addition, a cadaveric study investigating the surgical anatomy at risk with the dorsal approach found various structures at risk, including the extensor pollicis longus tendon, extensor indicis proprius tendon, extensor digitorum communis to the index, and terminal portion of the posterior interosseous nerve during dorsal guide wire insertion.[36]

In clinical practice, the volar approach has advantages over the dorsal approach because the volar approach can be used for fracture types at the waist and distal pole. Correction of a humpback deformity is easier using a volar approach because the wrist does not have to be flexed in contrast to a dorsal approach. Moreover, this approach carries little risk of damaging the main dorsal blood supply to the scaphoid.[22] However, the volar approach cannot achieve central screw placement without opening the scaphotrapezial joint, which can lead to degenerative changes and osteoarthritis, even though most patients do not experience symptoms.[12] Together, these findings suggest the need for another treatment option for acute scaphoid fractures. The transtrapezial approach may be a solution for such fractures. This treatment option may allow for more central screw placement at the distal pole of the scaphoid and increased biomechanical stability compared with the standard volar approach.[37]

The results of this meta-analysis did not support the theoretical advantage of the dorsal approach over the volar approach because all but one of the tested parameters, including nonunion rate, one of the most important parameters for assessing the clinical outcomes of fracture treatment, did not differ between the two approaches. Although the volar approach led to significantly greater ulnar deviation than the dorsal approach, the p-value was 0.05, just marginally significant. The less than 4 degree differences observed between the two approaches may have little clinical relevance and likely falls within the range of measurement error. Further studies are needed to determine whether these differences in fracture healing are clinically relevant. The similar outcomes for the two approaches may be explained by the fact that eccentric screw placement with the volar approach was less pronounced than expected, because various methods have been developed to diminish the trapezium interference and to achieve accurate central screw placement.[12] In addition, inappropriate measurement of screw length may have been more frequent with the dorsal approach than the volar approach. Screws placed through the dorsal approach could be shorter, resulting in less fixation strength because of the risk of cartilage damage at the radiocarpal joint and the shape of the proximal pole.

This study has several limitations. Of the seven studies, five were observational, resulting in some inherent heterogeneity due to uncontrolled bias, even though the studies had high quality scores. In addition, the heterogeneity of the included studies could be explained by slight differences in other factors affecting clinical outcomes, including the use of a wide variety of fixation devices and variability in fracture pattern. In detail, the results of B3 fractures are different compared with B1 and B2 fractures because the majority B3 fractures where treated using the dorsal approach which may negatively affect the overall results of this group of patients. However, the heterogeneities of the current studies were low, indicating that the pooled results of the current meta-analysis are reliable and robust across the domain of included studies.[38]

## Conclusions

This meta-analysis found no significant differences in the nonunion rate, postoperative complications, overall functional outcome, postoperative pain scale, grip strength, or range of wrist motion, including flexion, extension, and radial deviation, in patients who underwent surgical treatment using the dorsal or volar approach for acute scaphoid fracture. However, the volar approach resulted in significantly greater ulnar deviation than the dorsal approach. Thus, orthopedic surgeons need to master both the dorsal and volar percutaneous approaches because not all acute scaphoid fractures can be dealt with completely with one approach.

## Supporting Information

S1 Fig (PDF)

## References

[1] Suh N, Benson EC, Faber KJ, MacDermid J, Grewal R. Treatment of acute scaphoid fractures: A systematic review and meta-analysis. Hand. 2010;5(4):345–53. 10.1007/s11552-010-9276-6 22131912 PMC2988115

[2] Shen L, Tang J, Luo C, Xie X, An Z, Zhang C. Comparison of operative and non-operative treatment of acute undisplaced or minimally-displaced scaphoid fractures: A meta-analysis of randomized controlled trials. PloS one. 2015;10(5):e0125247 10.1371/journal.pone.0125247 25942316 PMC4420279

[3] Ibrahim T, Qureshi A, Sutton AJ, Dias JJ. Surgical versus nonsurgical treatment of acute minimally displaced and undisplaced scaphoid waist fractures: Pairwise and network meta-analyses of randomized controlled trials. J Hand surg Am. 2011;36(11):1759–68.e1. 10.1016/j.jhsa.2011.08.033 22036276

[4] Dias JJ, Wildin CJ, Bhowal B, Thompson JR. Should acute scaphoid fractures befixed?. J Bone Joint Surg Am. 2005;87(10):2160–8. 16203878 10.2106/JBJS.D.02305

[5] McQueen MM, Gelbke MK, Wakefield A, Will EM, Gaebler C. Percutaneous screw fixation led to faster recovery and return to work than immobilization for fractures of the waist of the scaphoid. J Bone Joint Surg Br. 2008;90:66–71. 18160502 10.1302/0301-620X.90B1.19767

[6] Shin AY, Hofmeister EP. Percutaneous fixation of stable scaphoid fractures. Tech Hand Up Extrem Surg. 2004;8(2):87–94. 16518119 10.1097/01.bth.0000129884.78204.a5

[7] Jeon IH, Micic ID, Oh CW, Park BC, Kim PT. Percutaneous screw fixation for scaphoid fracture: A comparison between the dorsal and the volar approaches. J Hand surg Am. 2009;34(2):228–36.e1. 10.1016/j.jhsa.2008.10.016 19181223

[8] Gürbüz Y, Kayalar M, Bal E, Toros T, Kucuk L, Sugun TS. Comparison of dorsaland volar percutaneous screw fixation methods in acute type B scaphoid fractures. Acta Orthop Traumatol Turc. 2012;46(5):339–45. 23268818

[9] Soubeyrand M, Biau D, Mansour C, Mahjoub S, Molina V, Gagey O. Comparison of percutaneous dorsal versus volar fixation of scaphoid waist fractures using acomputer model in cadavers. J Hand surg Am. 2009;34(10):1838–44. 10.1016/j.jhsa.2009.07.012 19969191

[10] Gehrmann SV, Grassmann JP, Wild M, Jungbluth P, Kaufmann RA, Windolf J, Hakimi M. Treatment of scaphoid waist fractures with the HCS screw. GMS Interdiscip Plast Recon Surg DGPW. 2014;3:Doc10. 10.3205/iprs000051 PMC4582507 26504721

[11] Chan KW, McAdams TR. Central screw placement in percutaneous screw scaphoid fixation: A cadaveric comparison of proximal and distal techniques. J Hand surg Am. 2004;29(1):74–9. 14751108 10.1016/j.jhsa.2003.09.002

[12] Meermans G, Van Glabbeek F, Braem MJ, van Riet RP, Hubens G, Verstreken F. Comparison of two percutaneous volar approaches for screw fixation of scaphoid waist fractures: radiographic and biomechanical study of an osteotomy-simulated model. J Bone Joint Surg Am. 2014;96(16):1369–76. 10.2106/JBJS.L.01729 25143497

[13] McCallister WV, Knight J, Kaliappan R, Trumble TE. Central placement of the screw in simulated fractures of the scaphoid waist: a biomechanical study. JBone Joint Surg Am. 2003;85-A(1): 72–7. 12533575 10.2106/00004623-200301000-00012

[14] Cooney WP, Dobyns JH, Linscheid RL. Fractures of the scaphoid: A rational approach to management. Clin Orthop Relat Res. 1980;149(149):90–7. 6996886

[15] Jester A, Harth A, Wind G, Germann G, Sauerbier M. Disabilities of the Arm, Shoulder and Hand (DASH) Questionnaire: Determining functional activity profilesin patients with upper extremity disorders. J Hand surg Br. 2005;30(1):23–8. 15620487 10.1016/j.jhsb.2004.08.008

[16] Parajuli NP, Shrestha D, Dhoju D, Shrestha R, Sharma V. Scaphoid Fracture: Functional outcome following fixation with Herbert screw. Kathmandu University Medical Journal. 2012;9(4):267–73. 10.3126/kumj.v9i4.6342 22710536

[17] Griffin D, Parsons N, Shaw E, Kulikov Y, Hutchinson C, Thorogood M, Lamb S. Operative versus non-operative treatment for closed, displaced, intra-articular fractures of the calcaneus: Randomised controlled trial. BMJ. 2014;349:g4483 10.1136/bmj.g4483 25059747 PMC4109620

[18] Wells GA, Shea B, O’connell D, Peterson J, Welch V, Losos M, Tugwell P. The Newcastle-Ottawa Scale (NOS) for assessing the quality of nonrandomised studies in meta-analyses. Available: http://www.ohri.ca/programs/clinical_epidemiology/oxford.asp.

[19] Deeks JJ, Higgins JPT, Altman DG, Green S. Cochrane handbook for systematicreviews of interventions version 5.1. 0 (Updated March 2011). The CochraneCollaboration (2011).

[20] Slade JF, Lozano-Calderón S, Merrell G, Ring D. Arthroscopic-assisted percutaneous reduction and screw fixation of displaced scaphoid fractures. J Hand surg Eur Vol. 2008;33(3):350–4. 10.1177/1753193408090121 18562371

[21] Drac P, Cizmar I, Manak P, Hrbek J, Reska M, Filkuka P, Zapletalova J. Comparison of the results and complications of palmar and dorsal miniinvasive approaches in the surgery of scaphoid fractures. A prospective randomized study. Biomed Pap Med Fac Univ Palacky Olomouc Czech Repub. 2012;158(2):277–81. 10.5507/bp.2012.060 23149467

[22] Polsky MB, Kozin SH, Porter ST, Thoder JJ. Scaphoid Fractures: Dorsal versusvolar approach. Orthopedics. 2002;25(8):817–9. 12195907 10.3928/0147-7447-20020801-11

[23] Drac P, Manak P, Cizmar I, Hrbek J, Zapletalová J. [a palmar percutaneous volarversus a dorsal limited approach for the treatment of non-and minimally-displaced scaphoid waist fractures: An assessment of functional outcomes and complications]. Acta Chir Orthop Traumatol Cech. 2010;77(2):143–8. 20447359

[24] Bushnell BD, McWilliams AD, Messer TM. Complications in dorsal percutaneous cannulated screw fixation of nondisplaced scaphoid waist fractures. J Hand surg Am. 2007;32(6):827–33. 17606062 10.1016/j.jhsa.2007.04.003

[25] Slade JF, Grauer JN, Mahoney JD. Arthroscopic reduction and percutaneous fixation of scaphoid fractures with a novel dorsal technique. Orthop Clin North Am. 2001;32(2):247–61. 11331539 10.1016/s0030-5898(05)70247-9

[26] Levitz S, Ring D. Retrograde (volar) scaphoid screw insertion-a quantitative computed tomographic analysis. J Hand Surg Am. 2005;30(3):543–8. 15925165 10.1016/j.jhsa.2004.12.014

[27] Leventhal EL, Wolfe SW, Walsh EF, Crisco JJ. A computational approach to the “optimal” screw axis location and orientation in the scaphoid bone. J Hand Surg Am. 2009;34(4):677–84. 10.1016/j.jhsa.2009.01.011 19345870

[28] Meermans G, Verstreken F. A comparison of 2 methods for scaphoid central screw placement from a volar approach. J Hand Surg Am. 2011;36(10):1669–74. 10.1016/j.jhsa.2011.06.023 21849237

[29] Menapace KA, Larabee L, Arnoczky SP, Neginhal VS, Dass GA, Ross LM. Anatomic placement of the Herbert-Whipple screw in scaphoid fractures: A cadaver study. J Hand surg Am. 2001;26(5):883–92. 11561242 10.1053/jhsu.2001.27755

[30] Kamineni S, Lavy CB. Percutaneous fixation of scaphoid fractures. An anatomical study. J Hand Surg Br. 1999;24(1): 85–8. 10190613 10.1016/s0266-7681(99)90043-8

[31] Botte MJ, Mortensen WW, Gelberman RH, Rhoades CE, Gellman H. Internal vascularity of the scaphoid in cadavers after insertion of the Herbert screw. JHand Surg Am. 1988;13(2):216–20. 3351245 10.1016/s0363-5023(88)80051-0

[32] Slade JF, Taksali S, Safanda J. Combined fractures of the scaphoid and distal radius: A revised treatment rationale using percutaneous and arthroscopic techniques. Hand clinics. 2005;21(3):427–41. 16039454 10.1016/j.hcl.2005.03.004

[33] Bedi A, Jebson PJ, Hayden RJ, Jacobson JA, Martus JE. Internal fixation of acute, nondisplaced scaphoid waist fractures via a limited dorsal approach: An assessment of radiographic and functional outcomes. J Hand surg Am. 2007;32(3):326–33. 17336838 10.1016/j.jhsa.2007.01.002

[34] Meermans G, Verstreken F. Influence of screw design, sex, and approach in scaphoid fracture fixation. Clin Orthop Relat Res. 2012;470(6):1673–81. 10.1007/s11999-011-2218-y 22179982 PMC3348297

[35] Dodds SD, Panjabi MM, Slade JF 3rd. Screw fixation of scaphoid fractures: abiomechanical assessment of screw length and screw augmentation. J Hand Surg Am. 2006;31(3):405–13. 16516734 10.1016/j.jhsa.2005.09.014

[36] Adamany DC, Mikola EA, Fraser BJ. Percutaneous fixation of the scaphoid through a dorsal approach: An anatomic study. J Hand surg Am. 2008;33(3):327–31. 10.1016/j.jhsa.2007.12.006 18343286

[37] Meermans G, Verstreken F. Percutaneous Transtrapezial fixation of acute scaphoidfractures. J Hand surg Eur Vol. 2008;33(6):791–6. 10.1177/1753193408092785 18694924

[38] Huedo-Medina TB, Sánchez-Meca J, Marín-Martínez F, Botella J. Assessing heterogeneity in meta-analysis: Q Statistic or I^2^ Index? Psychol Methods. 2006;11(2):193–206. 16784338 10.1037/1082-989X.11.2.193

